# Tumble Suppression Is a Conserved Feature of Swarming Motility

**DOI:** 10.1128/mBio.01189-20

**Published:** 2020-06-16

**Authors:** Jonathan D. Partridge, Nguyen T. Q. Nhu, Yann S. Dufour, Rasika M. Harshey

**Affiliations:** a Department of Molecular Biosciences, University of Texas at Austin, Austin, Texas, USA; b Department of Microbiology and Molecular Genetics, Michigan State University, East Lansing, Michigan, USA; University of Washington

**Keywords:** *Bacillus*, chemotaxis, *E. coli*, flagellar motility, Lévy walk, *Proteus*, *Pseudomonas*, *Salmonella*, *Serratia*, surface motility, swarming, tumble bias, *Bacillus subtilis*, *Escherichia coli*, *Proteus mirabilis*, *Pseudomonas aeruginosa*, *Salmonella enterica*, *Serratia marcescens*

## Abstract

Many bacteria use flagellum-driven motility to swarm or move collectively over a surface terrain. Bacterial adaptations for swarming can include cell elongation, hyperflagellation, recruitment of special stator proteins, and surfactant secretion, among others. We recently demonstrated another swarming adaptation in Escherichia coli, wherein the chemotaxis pathway is remodeled to decrease tumble bias (increase run durations), with running speeds increased as well. We show here that the modification of motility parameters during swarming is not unique to E. coli but is shared by a diverse group of bacteria we examined—Proteus mirabilis, Serratia marcescens, Salmonella enterica, Bacillus subtilis, and Pseudomonas aeruginosa—suggesting that increasing run durations and speeds are a cornerstone of swarming.

## Abstract (precis)

Bacteria within a swarm move characteristically in packs, displaying an intricate swirling motion in which hundreds of dynamic rafts continuously form and dissociate as the swarm colonizes an increasing expanse of territory. The demonstrated property of E. coli to reduce its tumble bias and hence increase its run duration during swarming is expected to maintain and promote side-by-side alignment and cohesion within the bacterial packs. In this study, we observed a similar low tumble bias in five different bacterial species, both Gram positive and Gram negative, each inhabiting a unique habitat and posing unique problems to our health. The unanimous display of an altered run-tumble bias in swarms of all species examined in this investigation suggests that this behavioral adaptation is crucial for swarming.

## OBSERVATION

Swarming is defined as a rapid collective migration of bacteria across a surface, powered by flagella and assisted by a wide array of phenotypic adaptations (1–3). A common attribute of all swarms is a pattern of ceaseless circling motion, in which packs of cells all traveling in the same directions split and merge, with continuous exchange of bacteria between the packs (3–5). This behavior differs from movement of the bacteria in bulk liquid, where they swim individually (6). In Escherichia coli, the mechanics of flagella are similar during both swimming and swarming in that peritrichous flagella driven by bidirectional rotary motors switch between counterclockwise (CCW) and clockwise (CW) directions. However, while CCW rotation promotes formation of a coherent flagellar bundle that propels the cell forward (run) during both swimming and swarming, a transient switch in rotational direction (CW) causes the cell to tumble while swimming but reverse direction while swarming (7, 8). There are variations on this theme. For example, the motility of Pseudomonas aeruginosa is characterized as a run-reverse-turn, where prolonged runs are interrupted by a reversal and “flick” to cause a change in direction (9).

The switching frequency of the flagellar motor is controlled by the chemotaxis system, best studied in E. coli, where transmembrane receptors detect extracellular signals and transmit them via phosphorelay to the motor, to promote migration to favorable locales during swimming (10). The ability to perform chemotaxis is not essential for swarming, but a basal tumble bias (TB) is important (11). We recently reported that E. coli cells taken from a swarm exhibit more highly extended runs and higher speeds than planktonic cells and that this low tumble bias is the optimal bias for maximizing swarm expansion (12). Posttranscriptional changes that alter the levels of a key signaling protein suggested that the chemotaxis signaling pathway is reprogrammed for swarming. A low tumble bias is consistent with the superdiffusive Lévy walk run trajectories observed in swarms of Serratia marcescens and Bacillus subtilis (13) and could improve swarming performance at the minimum by favoring the alignment of cells all travelling in the same direction in a pack. Whether bacteria still perform chemotaxis during swarming is not known, but an avoidance response was observed when antibiotics were added to the swarm media; this response was not to the antibiotics *per se* (14). Swarming allows bacteria opportunities for dispersal in ecological niches and contributes to pathogenicity in many species (15), notably in conferring enhanced resistance to antibiotics (14).

In this study, we examined TB and speeds during swarming in a selected mix of swarmer species, united only in their macroscopic display of swirling packs. The disparities between these bacteria relating to swarming behavior are many. To begin with, the bacteria are fastidious with respect to the consistency of the agar on which they swarm, and accordingly, they display different phenotypes. For example, Proteus mirabilis elongates substantially (10 to 80 μm) on hard agar (1.5% and above) (16) but not on softer agar. The other four bacteria swarm only on softer agar (0.5% to 0.8% agar). Flagellum arrangements in these bacteria also vary: P. aeruginosa has a polar flagellum (17), while the other bacteria are peritrichously flagellated. P. mirabilis is substantially hyperflagellated on hard agar (16) and B. subtilis and P. aeruginosa double their flagellum numbers (17, 18), while Salmonella enterica and S. marcescens do not substantially change these numbers (3). S. enterica does not secrete surfactants or polysaccharides that lubricate the surface; the others all do. Despite these various swarming adaptations, we found that these bacteria all share the same low TB and higher run speeds as reported for E. coli, suggesting that this behavior is a universal adaptation for successful migration on a surface.

The methodology and growth conditions used to monitor TB and speed in this study were similar to those used for E. coli (12) and were consistently applied across all swarming species. Eiken agar was used to solidify swarm media, because this agar facilitates swarming in non-surfactant producers (19). By using the same agar concentration for all species (0.5%), we kept P. mirabilis from elongating; long cells do not tumble. Under these conditions, the cell length of this bacterium during swarming was similar to that of all the other species (2.5 ± 0.7 μm; *n *= 50), unchanged from that observed in liquid (2.1 ± 0.5 μm; *n *= 50). Preliminary tracking experiments with S. marcescens cells taken from liquid showed large circular trajectories (Fig. S1, left). Such trajectories have been observed with E. coli and Caulobacter crescentus swimming close to a glass surface (20). We suspected that the surfactant serrawettin, a cyclic lipopeptide secreted by S. marcescens (3), might be responsible for this behavior by suppressing tumbles. A S. marcescens mutant deficient in serrawettin production abolished the circular motion (Fig. S1, right), so this strain was used for tracking. B. subtilis makes a surfactant similar to serrawettin (2) and also displayed circular trajectories, so we used a *srfA* mutant deficient in surfactin synthesis. P. aeruginosa surfactant has a different structure (rhamnolipid) (2) and did not show such trajectories, so we worked with the wild-type strain. We discuss our findings in the order of discovery of swarming in the bacterial species studied in this investigation (21–25).

10.1128/mBio.01189-20.1 FIG S1 Representative trajectories of wild-type Serratia marcescens and RH1041, a serrawettin-deficient mutant (SMu4e in reference 28). Cells were grown in LB (liquid) plus glucose (0.5%, wt/vol) before transfer to LB liquid for observation. Cell movement was recorded for 100 s using phase-contrast microscopy at a magnification of ×10. Trajectories of single representative experiments shown. Different colors correspond to individual tracks. Download FIG S1, TIF file, 1.4 MB. Copyright © 2020 Partridge et al. 2020 Partridge et al. This content is distributed under the terms of the Creative Commons Attribution 4.0 International license.

**FIG 1:**
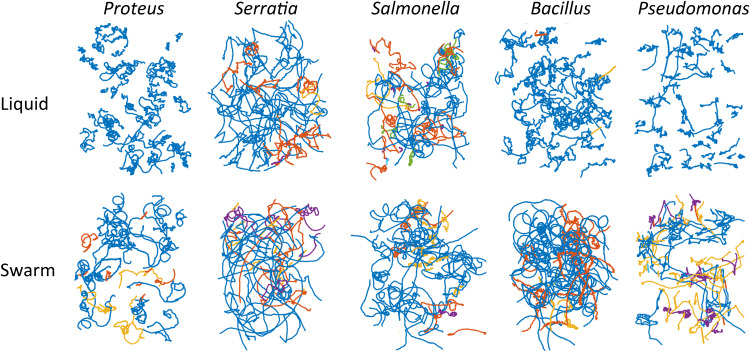
Trajectories of *Proteus*, *Serratia*, *Salmonella*, *Bacillus*, and *Proteus* cells cultivated under liquid or swarm conditions. Cells were grown in LB (liquid) or LB swarm agar, each supplemented with glucose (0.5%, wt/vol), before transfer to LB liquid for observation in a pseudo-2-dimensional (pseudo-2D) environment. Swarm plates were solidified with 0.5% Eiken agar (12). Cell movement was recorded for 100 s using phase-contrast microscopy at a magnification of ×10. Trajectories in single representative experiments are shown. Different colors correspond to individual tracks. *Salmonella enterica* 14028 and *Serratia marcescens* 274 were sourced from the American Type Culture Collection, *S. marcescens* serrawettin^−^ (RH1041; see Fig. S1), *Bacillus subtilis* *srfA* (DS191; gift from Daniel Kearns), *Pseudomonas aeruginosa* (PA01; gift from Verinita Gordon), and *Proteus mirabilis* (lab collection).

**FIG 2:**
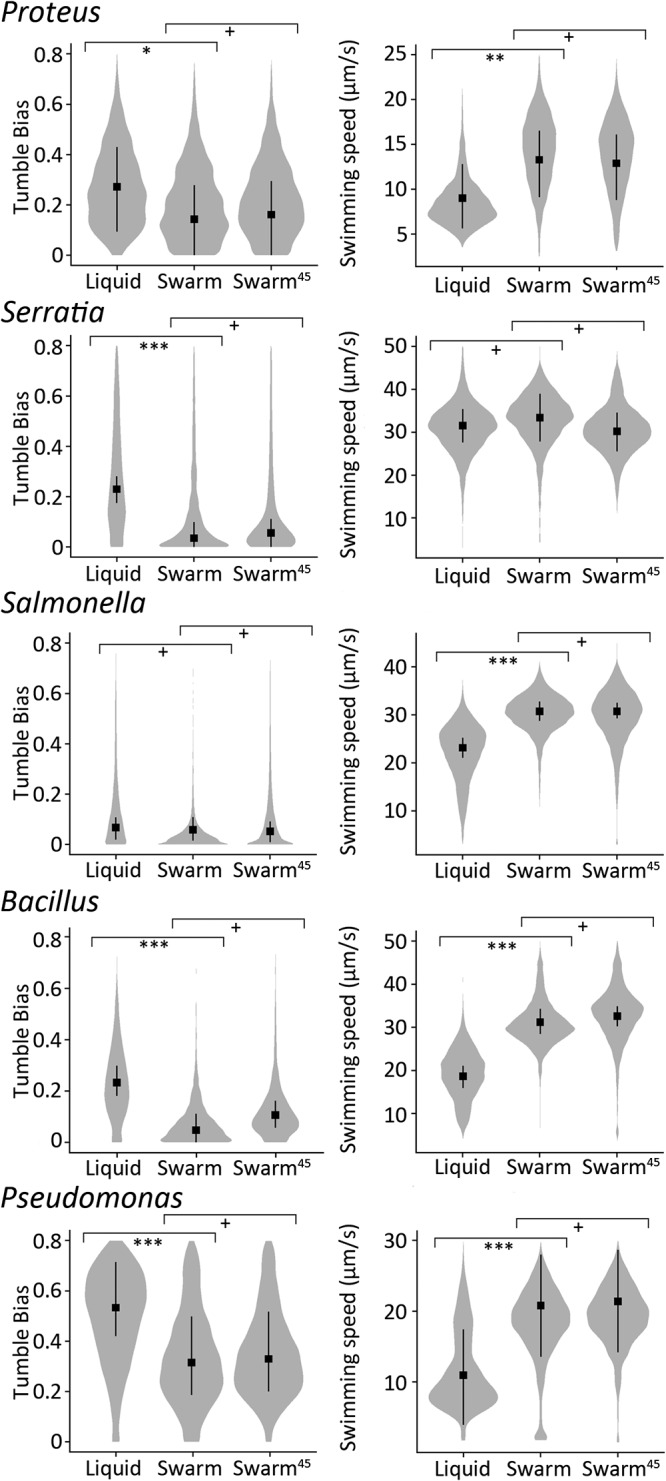
Tumble bias and swimming speeds of *Proteus*, *Serratia*, *Salmonella*, *Bacillus*, and *Pseudomonas* cells cultivated in liquid, swarm, or swarm^45^ conditions. Cells were grown in LB (liquid) or LB swarm agar, each supplemented with glucose (0.5%, wt/vol), before transfer to LB liquid for observation in a pseudo-2D environment. “Swarm^45^” indicates isolated “swarm” samples monitored again after 45 min had elapsed. Cell movement was recorded for 100 s using phase-contrast microscopy at a magnification of ×10. Probability distribution of cell tumble biases (left) and swimming speeds (right) are shown. The distribution of each parameter was calculated from more than 4,600 individual trajectories (>1,000 min of cumulative time) for each condition, from at least three independent experiments. The square and bars indicate the mean and 95% credible intervals of the posterior probabilities of the medians for each treatment. Calculated *P* values are indicated as follows: *, <0.05; **, <0.01; ***, <0.0001; and +, >0.05.

Representative cell trajectories in liquid and swarm media for all bacterial species tested are shown in Fig. 1. All show a distinct shift in motion paths under the two conditions, becoming smoother (long run trajectories) during swarming. Quantitative analyses of these trajectories are shown in Fig. 2. While technically P. aeruginosa does not tumble, in our analysis, the run-reverse and reverse-flick were both identified as tumbles. The tumble angle distribution plots generated were consistent with run-reverse-flick. The changes in median TB values from liquid to swarm were as follows: P. mirabilis, 0.27 to 0.14; S. marcescens, 0.23 to 0.037; S. enterica, 0.07 to 0.05; B. subtilis, 0.24 to 0.048; and P. aeruginosa, 0.53 to 0.31 (statistics are detailed in Table S1). While the overall pattern was that TBs shifted to lower values during swarming, we note that TB values for S. enterica are lower than for E. coli in liquid to begin with, as reported in single motor assays (26). For comparison, TB values for E. coli decreased from a median of 0.12 in liquid to 0.04 in swarmers (12).

10.1128/mBio.01189-20.2 TABLE S1 Mean posterior probabilities for the median tumble biases and swimming speeds and comparisons of *Proteus*, *Serratia*, *Salmonella*, *Bacillus*, and *Pseudomonas* cells cultivated under liquid, swarm, or swarm^45^ conditions. Bayesian sampling was used to determine if the medians of the swimming speed and tumble bias were significantly different between liquid, swarm, and swarm^45^ preparations. “Swarm^45^” indicates isolated “swarm” samples monitored again after 45 min had elapsed. The posterior probability distributions of the medians for each strain and each treatment were calculated using a linear mixed-effect model ((Swimming_speed, Tumble_bias) ~ Treatment + 1|Replicate) with a Gaussian distribution link function. All the statistical analyses were done by sampling of the respective mixed-effect generalized linear models using the RSTAN (29) and BRMS packages (30) in R (31) with 4 chains, each with 1,000 warmup iterations and at least 5,000 sampling iterations. *P* values and credible intervals were calculated by sampling the posterior probability distributions. Uninformative priors were set to the defaults generated by BRMS. The plots were generated using the ggplot2 (32) and tidybayes (33) packages. The mean and 95% credible intervals^a^ of the posteriors of the medians for each distribution are also reported. The means and 95% credible intervals^b^ of the differences of the medians between conditions is reported. *P* values^c^ (for difference in the medians of >0 or <0) were calculated by sampling the posterior probability distributions. Download Table S1, DOCX file, 0.02 MB. Copyright © 2020 Partridge et al. 2020 Partridge et al. This content is distributed under the terms of the Creative Commons Attribution 4.0 International license.

The low TB displayed by E. coli swarmers was observed to be stable for up to 45 min and persisted through one cell division at room temperature (∼120 min); this physiological adaptation is different from the adaptation of the chemotaxis pathway through methylation (12). We therefore also included a 45-min time point (after lifting cells from the swarm) for tracking all five swarmers. At 45 min after removal from the swarm, most bacteria maintained their low TB values (statistics in Table S1).

As observed for E. coli, running speeds (micrometers per second) for a majority of the bacterial species increased significantly between liquid and swarm as follows: P. mirabilis, 9.01 to 13.3; S. enterica, 23.1 to 30.7; B. subtilis, 18.6 to 31; and P. aeruginosa, 21.9 to 41.6 (statistics in Table S1). These values for E. coli were 21 μm/s in liquid and 25 μm/s in swarmers (12).

In summary, keeping swarming conditions the same, we demonstrated that despite different natural habitats and widely different swarming adaptations discovered in the laboratory, the swarmers studied all modify their TB, and a majority modify run speeds during swarming, similar to what was reported for E. coli (12). This apparently common behavior suggests that it represents a successful strategy for collective migration across a surface. There are many ways to reduce TB and increase speed, and the precise mechanisms of altering these parameters may vary in the different species. In E. coli, elevation or stabilization of the chemotaxis component CheZ is responsible for the low TB (12). Other components in the chemotaxis pathway can potentially be altered to achieve the same outcome. As demonstrated for S. marcescens, surfactants themselves contribute to lowering TB (Fig. S1), although our experiments bypassed the surfactant. Swarming speed can be increased by hyperflagellation, torque-enhancing proteins, special stators, and increased proton motive force (3, 27). Future work will reveal the mechanisms used by each of these bacteria to arrive at what is apparently a common solution for maximizing collective motion.

## 

### Methods.

Cell culture, swarm setup, tracking experiments, and analysis were largely carried out as described previously (12).
