# Supplementary figures and images for: Tumble Suppression Is a Conserved Feature of Swarming Motility
Source: mBio. 2020 Jun 16;11(3):e01189-20. doi: 10.1128/mBio.01189-20 (PMC7298715; doi:10.1128/mBio.01189-20)

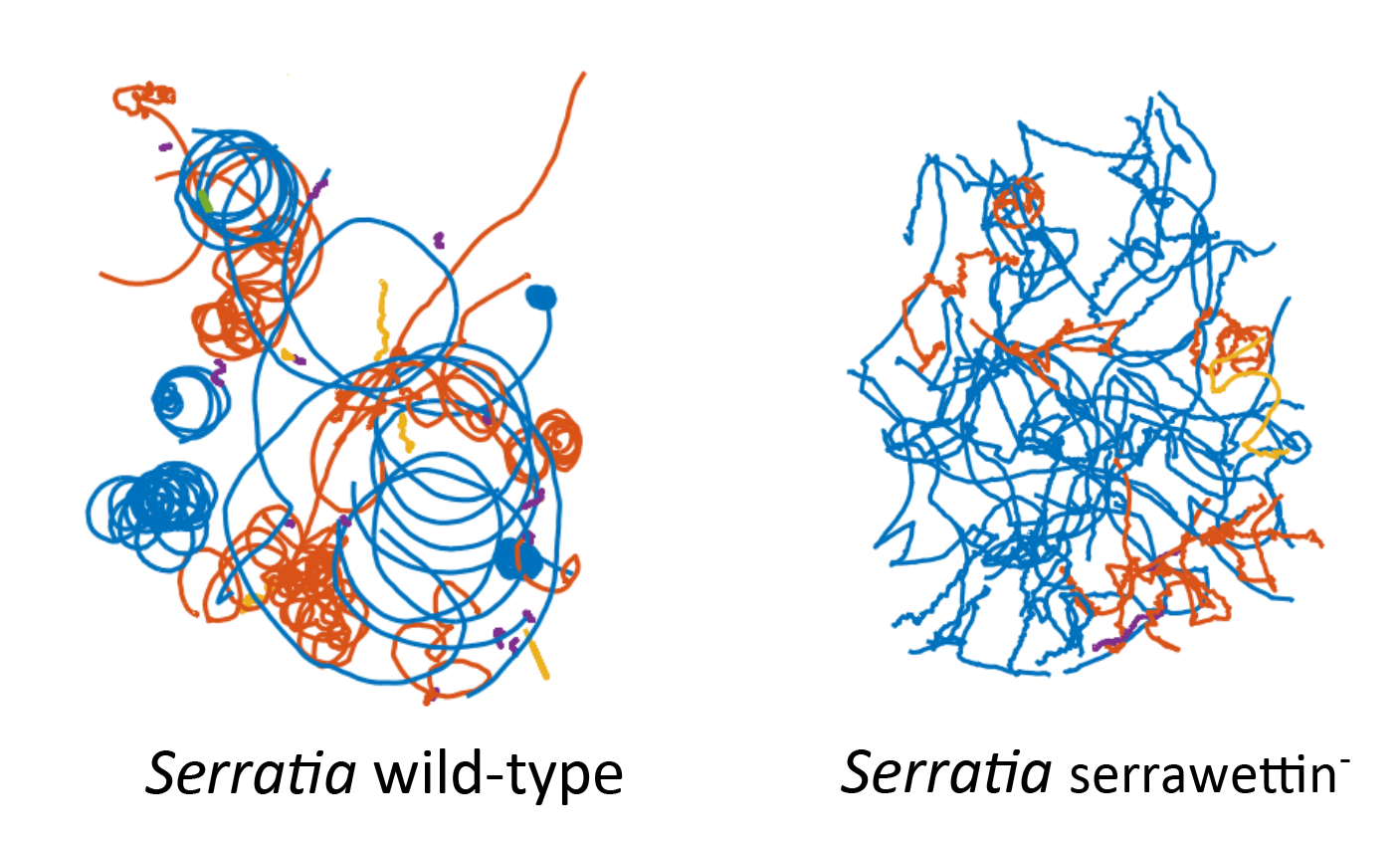

Supplement: FIG S1 [file mBio.01189-20-sf001.tif]
