# Supplementary material for: Tumble Suppression Is a Conserved Feature of Swarming Motility
Source: mBio. 2020 Jun 16;11(3):e01189-20. doi: 10.1128/mBio.01189-20 (PMC7298715; doi:10.1128/mBio.01189-20)
Supplement: TABLE S1 [file mBio.01189-20-st001.docx]

**Table S1**

|  | **Median posteriors^a^ [95% CI]** |  | **Conditions contrast^b^ [95% CI]** | ***P* value^c^** |
| --- | --- | --- | --- | --- |
| ***Proteus* Speeds** |  |  |  |  |
| **Liquid** | 9.01 [5.63, 12.81] | Liquid vs. Swarm | -4.28 [-7.55, -1.59] | 2.4E-3 |
| **Swarm** | 13.29 [9.10, 16.52] | Swarm vs. Swarm^45^ | -0.39 [-1.88, 1.11] | 2.2E-1 |
| **Swarm^45^** | 12.90 [8.78, 16.10] | Liquid vs. Swarm^45^ | -3.89 [-7.13, -1.19] | 4.1E-3 |
| ***Proteus* TB** |  |  |  |  |
| **Liquid** | 0.27 [0.09, 0.42] | Liquid vs. Swarm | 0.13 [-0.01, 0.27] | 3.1E-2 |
| **Swarm** | 0.14 [0, 0.28] | Swarm vs. Swarm^45^ | 0.02 [-0.05, 0.09] | 2.4E-1 |
| **Swarm^45^** | 0.16 [0, 0.29] | Liquid vs. Swarm^45^ | 0.11 [-0.02, 0.25] | 4.6E-2 |
| ***Serratia* Speeds** |  |  |  |  |
| **Liquid** | 31.56 [27.59, 35.37] | Liquid vs. Swarm | -1.85 [-7.16, 3.46] | 2.3E-1 |
| **Swarm** | 33.41 [27.86, 38.98] | Swarm vs. Swarm^45^ | -3.22 [-8.75, 2.51] | 1.2E-1 |
| **Swarm^45^** | 30.18 [25.50, 34.60] | Liquid vs. Swarm^45^ | 1.37 [-2.89, 5.61] | 2.4E-1 |
| ***Serratia* TB** |  |  |  |  |
| **Liquid** | 0.23 [0.17, 0.28] | Liquid vs. Swarm | 0.19 [0.14, 0.25] | <1E-05 |
| **Swarm** | 0.04 [0, 0.10] | Swarm vs. Swarm^45^ | 0.02 [-0.03, 0.08] | 2.0E-1 |
| **Swarm^45^** | 0.06 [0, 0.11] | Liquid vs. Swarm^45^ | 0.19 [0.14, 0.25] | <1E-05 |
| ***Salmonella* Speeds** |  |  |  |  |
| **Liquid** | 23.12 [21.02, 25.19] | Liquid vs. Swarm | -7.59 [-9.84, -5.36] | <1E-05 |
| **Swarm** | 30.71 [29.24, 32.47] | Swarm vs. Swarm^45^ | -0.01 [-2.41, 2.26] | 5.0E-1 |
| **Swarm^45^** | 30.70 [28.75, 32.72] | Liquid vs. Swarm^45^ | -7.58 [-10.39, -4.84] | 1.4E-04 |
| ***Salmonella* TB** |  |  |  |  |
| **Liquid** | 0.07 [0.02, 0.11] | Liquid vs. Swarm | 0.02 [-0.00, 0.04] | 6.7E-2 |
| **Swarm** | 0.05 [0.01, 0.09] | Swarm vs. Swarm^45^ | 0.01 [-0.04, 0.037] | 3.1E-1 |
| **Swarm^45^** | 0.06 [0.02, 0.11] | Liquid vs. Swarm^45^ | 0.01 [-0.03, 0.07] | 4.1E-1 |
| ***Bacillus* Speeds** |  |  |  |  |
| **Liquid** | 18.64 [15.87, 21.10] | Liquid vs. Swarm | -12.57 [-15.87, -8.83] | <1E-05 |
| **Swarm** | 31.20 [28.43, 34.31] | Swarm vs. Swarm^45^ | 1.37 [-1.37, 4.78] | 2.1E-1 |
| **Swarm^45^** | 32.58 [30.26, 34.86] | Liquid vs. Swarm^45^ | -13.94 [-15.74, -11.69] | <1E-05 |
| ***Bacillus* TB** |  |  |  |  |
| **Liquid** | 0.23 [0.18, 0.30] | Liquid vs. Swarm | 0.18 [0.11, 0.27] | 2.5E-4 |
| **Swarm** | 0.048 [0, 0.11] | Swarm vs. Swarm^45^ | 0.06 [-0.02, 0.13] | 5.0E-2 |
| **Swarm^45^** | 0.11 [0.06, 0.16] | Liquid vs. Swarm^45^ | 0.13 [0.07, 0.18] | 6.5E-4 |
| ***Pseudomonas* Speeds** |  |  |  |  |
| **Liquid** | 21.91 [7.92, 34.90] | Liquid vs. Swarm | -19.70 [-28.33, -9.76] | 9.0E-04 |
| **Swarm** | 41.60 [27.10, 55.96] | Swarm vs. Swarm^45^ | 1.14 [-8.66, 11.02] | 4.0E-1 |
| **Swarm^45^** | 42.75 [28.41, 57.34] | Liquid vs. Swarm^45^ | -20.83 [-29.60, -10.58] | 7.0E-4 |
| ***Pseudomonas* TB** |  |  |  |  |
| **Liquid** | 0.53 [0.42, 0.71] | Liquid vs. Swarm | 0.22 [0.13, 0.30] | 3.0E-4 |
| **Swarm** | 0.31 [0.19, 0.50] | Swarm vs. Swarm^45^ | 0.015 [-0.08, 0.11] | 3.7E-1 |
| **Swarm^45^** | 0.33 [0.20, 0.52] | Liquid vs. Swarm^45^ | 0.20 [0.11, 0.29] | 1.0E-3 |

**Table S1. Mean posterior probabilities for the median tumble biases and swimming speeds and their comparisons of *Proteus, Serratia, Salmonella, Bacillus,* and *Pseudomonas* cells cultivated in liquid, swarm, or swarm^45^ conditions.** Bayesian sampling was used to determine if the medians of the swimming speed and tumble bias are significantly different between liquid, swarm, and swarm^45^ preparations. Swarm^45^ denotes isolated ‘swarm’ samples monitored again after 45 min had elapsed. The posterior probability distributions of the medians for each strain and each treatment were calculated using a linear mixed-effect model with a Gaussian distribution link function. The mean and 95% credible intervals^a^ of the posteriors of the medians for each distribution is also reported. See supplementary information for more details. The means and 95% credible intervals^b^ of the differences of the medians between conditions is reported. *P* values^c^ (for difference in the medians >0 or <0) were calculated by sampling the posterior probability distributions.
